# Cardiac transthyretin amyloidosis in aortic valve replacement: RAISE score performance in the postoperative setting

**DOI:** 10.1007/s00392-025-02766-6

**Published:** 2025-11-04

**Authors:** Richard J. Nies, Svenja Ney, Jasper F. Nies, Katharina Seuthe, Jan Grobecker, Friedrich Gruenagel, Stephan Nienaber, Merve Kural, Sascha Macherey-Meyer, Matthieu Schäfer, Clemens Metze, Matti Adam, Maria Papathanasiou, Can Öztürk, Amin Polzin, Fabian Voß, Stephan Baldus, Roman Pfister

**Affiliations:** 1 University of Cologne, Faculty of Medicine and University Hospital Cologne, Clinic III for Internal Medicine, Kerpener Str. 62, 50937 Cologne, Germany; 2 Department II of Internal Medicine and Center for Molecular Medicine Cologne, Faculty of Medicine, University of Cologne, and University Hospital Cologne, Kerpener Str. 62, 50937 Cologne, Germany; 3 Department of Cardiology, University Hospital Frankfurt, Theodor-Stern-Kai 7, 60596 Frankfurt, Germany; 4 University Hospital Bonn, Heart Centre Bonn, Venusberg-Campus 1, 53127 Bonn, Germany; 5 Heart Centre Düsseldorf, University Hospital Düsseldorf, Moorenstr. 5, 40225 Düsseldorf, Germany

**Keywords:** Cardiac transthyretin amyloidosis, Aortic stenosis, Aortic valve replacement, RAISE Score, Cardiomyopathy

## Abstract

**Aims:**

The RAISE Score, encompassing five domains (*R*emodeling, *A*ge, *I*njury, *S*ystemic, *E*lectrical), was proposed to screen for transthyretin amyloid cardiomyopathy (ATTR-CM) in patients with aortic stenosis (AS), but is not routinely used. This study evaluated its performance following aortic valve replacement (AVR).

**Methods:**

This single-center, prospective, observational study included patients aged ≥ 60 years with end-diastolic interventricular septum thickness (IVSd) ≥ 12 mm, who underwent hydroxydiphosphonate (HDP) bone scintigraphy after AVR between March 2021 and July 2024. The diagnostic performance of the RAISE Score assessed at 30-day follow-up and of simpler screening parameters for pathological HDP uptake (Perugini 1–3) were analyzed, along with their association with all-cause mortality and heart failure (HF) hospitalization.

**Results:**

Among 131 included patients (median age 81 years; 64% male) pathological HDP uptake was found in 21 (16%), and 11 (8.4%) were diagnosed with ATTR-CM. A RAISE Score ≥ 2 demonstrated 76% sensitivity and 56% specificity for pathological bone scintigraphy, while a Score ≥ 3 showed 62% sensitivity and 78% specificity. The parameters age ≥ 83 years and the combination of carpal tunnel syndrome (CTS) and/or N-terminal pro-B-type natriuretic peptide (NT-proBNP) ≥ 1,400 pg/mL performed similarly to the RAISE Score. Cardiac HDP uptake showed a strong trend toward HF hospitalization (HR [95%-CI]: 5.81 [0.93–36.20]; p = 0.060) and all-cause mortality (HR [95%-CI]: 3.27 [0.95–11.24]; p = 0.060). CTS and/or NT-proBNP ≥ 1,400 pg/mL was significantly associated with higher all-cause mortality (HR [95%-CI]: 3.92 [1.04–14.84]; p = 0.044), whereas the RAISE Score was not.

**Conclusion:**

After AVR, the complex RAISE Score showed lower sensitivity than originally reported, while simpler parameters demonstrated comparable predictive value for ATTR-CM screening. Advanced age, CTS history and elevated NT-proBNP levels seem most valuable for identifying candidates for bone scintigraphy.

**Graphical Abstract:**

A total of 131 patients aged ≥ 60 years, with an IVSd ≥ 12 mm and an AVR between March 2021 and July 2024, underwent standard diagnostic evaluation for ATTR-CM, including HDP bone scintigraphy. Pathological findings on bone scintigraphy (Perugini Score 1–3) were observed in 16% of the patients and were associated with worse clinical outcomes. The performance of the proposed RAISE Score [11], along with simplified or modified screening parameters, was evaluated in predicting both pathological scintigraphy results and clinical outcomes. ATTR-CM = transthyretin amyloidosis cardiomyopathy; AVR = aortic valve replacement; CTS = carpal tunnel syndrome; HDP = hydroxydiphosphonate; IVSd = interventricular septum thickness at end-diastole; NT-proBNP = N-terminal pro-B-type natriuretic peptide.

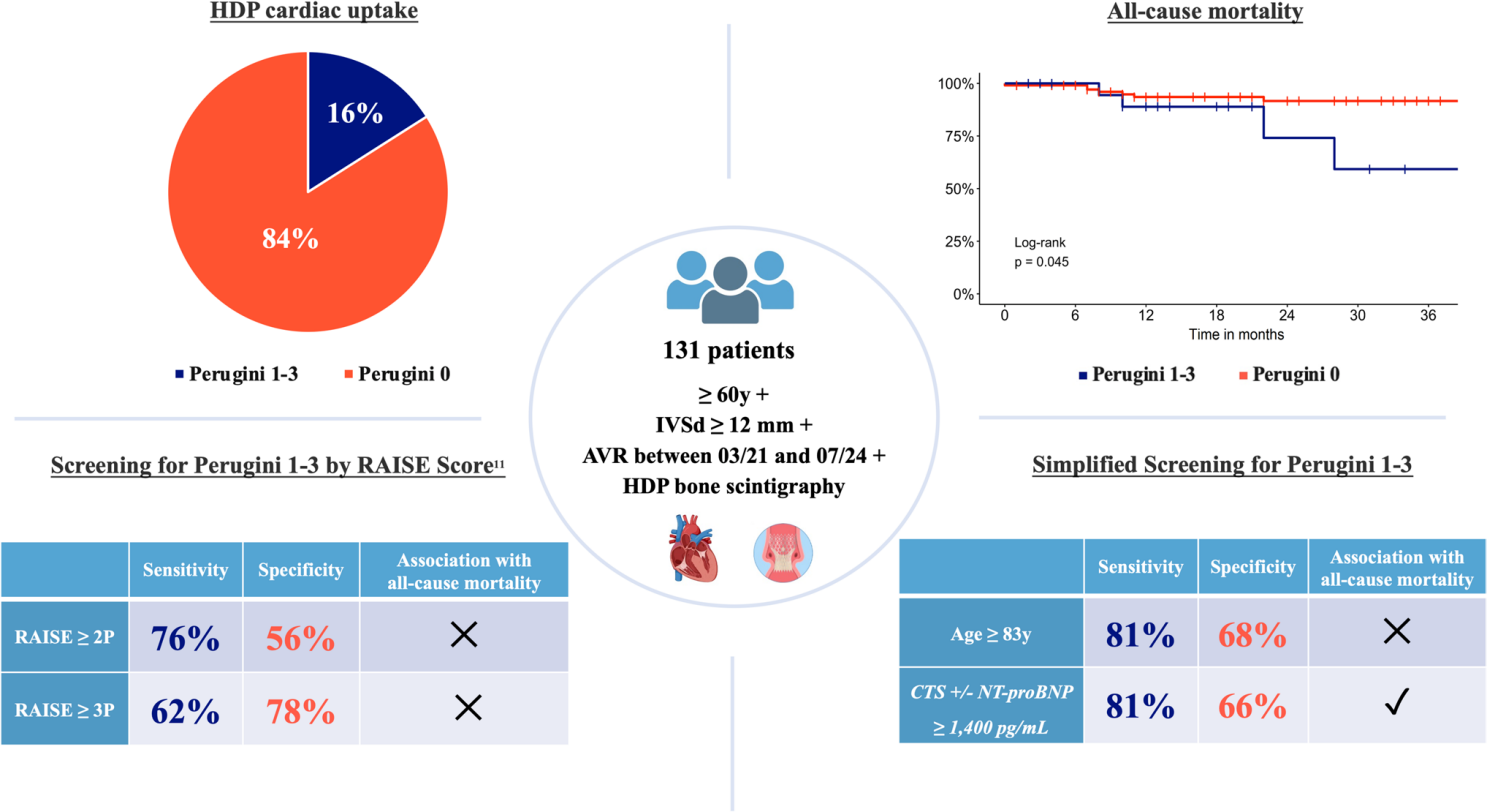

**Supplementary Information:**

The online version contains supplementary material available at 10.1007/s00392-025-02766-6.

## Introduction

Transthyretin amyloidosis cardiomyopathy (ATTR-CM) is a progressive infiltrative disease caused by myocardial deposition of misfolded transthyretin (TTR), with increasing clinical relevance due to advances in diagnostics and emerging therapies [1–6]. Early detection remains crucial, as current therapies can slow disease progression but cannot reverse amyloid deposition [3, 5, 6]. However, approximately half of patients with ATTR-CM are still diagnosed at advanced disease stages [7, 8].

To improve early diagnosis of ATTR-CM, clinical red flags should prompt further diagnostic evaluation in patients with left ventricular hypertrophy (LVH) ≥ 12 mm [1]. Aortic stenosis (AS) in individuals aged ≥ 65 years is one such red flag, as the prevalence of ATTR-CM is approximately 15% among patients with AS [9–11]. Given that AS affects over 3% of patients ≥ 75 years, a large cohort potentially at risk for ATTR-CM exists, highlighting the need for pre-selection [12]. The RAISE Score was developed to identify patients with AS who are likely to have ATTR-CM, by incorporating clinical and diagnostic parameters related to *R*emodeling, *A*ge, *I*njury, *S*ystemic, and *E*lectrical abnormalities before aortic valve replacement (AVR) [11]. Depending on the selected threshold (2 or 3), the RAISE Score has a sensitivity of 72% to 94% and a specificity of 52% to 84% for detecting patients with cardiac uptake in bone scintigraphy [11].

However, the complexity of the RAISE Score, comprising seven variables, limits its use in routine clinical practice and lacks external validation [11]. Moreover, screening before AVR is suboptimal, as periprocedural complications can alter patients’ health status and functional capacity, both key factors in assessing eligibility for costly ATTR-CM therapy [13–15]. Therefore, screening for ATTR-CM after AVR may be more appropriate, but the RAISE Score has not been evaluated in this setting, and post-interventional biomarker or electrocardiogram (ECG) changes could impact its predictive value.

This study aimed to: i) evaluate the sensitivity, specificity, and predictive accuracy of the RAISE Score in the post-AVR setting; ii) identify simple, objective, and most valuable pre-selection criteria for ATTR-CM screening in a post-AVR cohort; iii) analyze clinical outcomes based on bone scintigraphy results, the RAISE Score, and simplified pre-selection variables.

## Methods

### Study population and design

In this single-center, prospective observational study, we screened all patients who underwent transcatheter aortic valve replacement (TAVR) or a surgical aortic valve replacement (SAVR) due to severe AS between March 1st, 2021, and July 31st, 2024 for study eligibility. In line with recent recommendations of the European Society of Cardiology (ESC) for management of cardiomyopathies, inclusion criteria were age ≥ 60 years, an end-diastolic interventricular septum thickness (IVSd) ≥ 12 mm, and AVR performed at our Heart Centre [16]. Exclusion criteria included a home distance greater than 100 km from our Heart Centre precluding further follow-up (FU) examinations, inability to provide consent, and lack of sufficient information on imaging and biomarkers. Screening was conducted weekly, with a maximum of five eligible patients per week. For the routine 30-day FU after AVR, eligible patients were scheduled at our amyloidosis outpatient clinic for assessment of a detailed medical history, clinical examination, ECG, laboratory diagnostics (including serum and urine immunofixation and free light chain measurements), transthoracic echocardiography (TTE), functional assessments, and study enrollment. The ECGs from the 30-day FU were also compared with those performed before AVR. A bone scintigraphy was scheduled, and the results were categorized according to the Perugini Score (0–3) [16]. Only patients who underwent bone scintigraphy were included in the final study cohort.

Diagnosis of ATTR-CM was made in accordance with the recommendations of the ESC Cardiac Amyloidosis Working Group and the 2023 ESC Guidelines for the Management of Cardiomyopathies [1, 2]. In cases where the Perugini Score was 1, an endomyocardial biopsy (EMB) was recommended to the patient [16]. FU was assessed six months after inclusion of the last patient. All analyses were conducted using anonymized data, with informed consent obtained from all participants.

### Data collection

Clinical baseline and FU data were systematically collected, anonymized, and stored in a RedCap database. The decision and choice of AS intervention were made by the institutional Heart Team, consisting of experienced cardiologists, cardiac surgeons, and anaesthesiologists. TTE was performed in accordance with current guidelines from the American Society of Echocardiography [17, 18]. The RAISE Score was applied as described by Nitsche et al. and calculated at the 30-day FU [11]. The original Score was developed and named based on five key domains: *R*emodeling (LVH and/or significant diastolic dysfunction), *A*ge, *I*njury (high-sensitivity troponin T [hs-TnT]) levels), *S*ystemic involvement (carpal tunnel syndrome [CTS]), and *E*lectrical abnormalities (right bundle branch block [RBBB] or low voltages). The points assigned to each item are presented in Table 1. Cutoff values of an overall RAISE Score ≥ 2 and ≥ 3 points have been proposed as indicative of ATTR-CM [11]. Scintigraphy with thoracic Single-Photon Emission Computed Tomography (SPECT) was performed using a tracer of 99mTechnetium-hydroxyethylene diphosphonate (99mTc-HDP) [19]. The results were independently interpreted by local nuclear medicine specialists. Data on hospitalization and vital status were collected from clinical records, telephone interviews, and reports from primary care physicians.

**Table 1:** Scoring system according to the RAISE Score, adapted from Nitsche et al. [11]

Parameter	Points
CTS	3
RBBB	2
Age ≥ 85 years	1
Hs-TnT > 0.020 ng/ml	1
IVSd ≥ 18 mm	1
E/A ratio > 1.4 (if in SR)	1
Sokolow-Lyon-Index < 1.9 mV OR all limb leads with QRS amplitude ≤ 0.5 mV (if no BBB or PM)	1

*BBB *bundle branch block, *CTS *carpal tunnel syndrome, *Hs-TnT* high-sensitive troponin T, *IVSd *interventricular septum thickness at end-diastole, *PM* pacemaker, *RBBB *right bundle branch block, *SR *sinus rhythm

### Clinical outcomes

We analyzed clinical outcomes based on all-cause mortality and hospitalization due to heart failure (HF).

### Statistics

Data are reported as counts (percentages), means (standard deviations [SD]), or medians (interquartile ranges [IQR]), depending on the data type. The study cohort was divided into groups based on bone scintigraphy findings: normal (Perugini Score 0) and pathological (Perugini Score 1–3). Patient characteristics were compared between those with and without pathological bone scintigraphy using the unpaired t-test, Mann–Whitney U test, chi-square test, or Fisher’s exact test, as appropriate. For dependent samples assessed before and after AVR, the McNemar test and the Wilcoxon test were used, respectively. Normality of data distribution was assessed using the Kolmogorov–Smirnov or Shapiro–Wilk tests.

Regarding the RAISE Score, sensitivity was calculated as the proportion of true positives among all patients with the condition, while specificity was the proportion of true negatives among all patients without the condition. Positive predictive value (PPV) was determined as the proportion of true positives among all positive test results, and negative predictive value (NPV) was the proportion of true negatives among all negative test results. Calculations were performed for RAISE Score cutoff values of 2 and 3, respectively.

Variables with significant differences between patients with and without any pathological cardiac HDP uptake were identified as potential predictors of ATTR-CM. Using a Receiver Operating Characteristic (ROC) analysis, optimal thresholds of continuous variables were determined for definitive diagnosis of ATTR-CM, i.e., Perugini Score of ≥ 2 in the absence of monoclonal gammopathy of undetermined significance (MGUS).

Non-inferiority analyses of the simplified pre-selection criteria for pathological bone scintigraphy, compared to the RAISE Score thresholds, were performed using a non-inferiority margin of 0.1. The association of bone scintigraphy results*,* the RAISE Score, and the simplified pre-selection criteria with all-cause mortality and HF hospitalization were analyzed using Kaplan–Meier curves and the Log-rank test. Univariate Cox regression analyses were conducted to calculate hazard ratios (HR) with corresponding 95% confidence intervals (CI). A two-sided *p-*value of < 0.05 was considered statistically significant in all tests. Statistical analyses were performed using IBM SPSS Statistics, version 29.0.0.0. ROC analyses were conducted using RStudio (version 2023.06.2 + 561) with the pROC (version 1.18.5), ROCR (version 1.0–11), and foreign (version 0.8–87) packages. Kaplan–Meier survival plots were generated in R (version 4.2.0) using the UpSetR (version 1.4.0), survival (version 3.3–1), and survminer (version 0.4.9) packages. For additional graphical illustrations, Microsoft Excel for Mac (version 16.96.1) and Microsoft PowerPoint for Mac (version 16.96.1) were used.

## Results

### Study population and prevalence of pathological bone scintigraphy and ATTR-CM

**Fig. 1:**
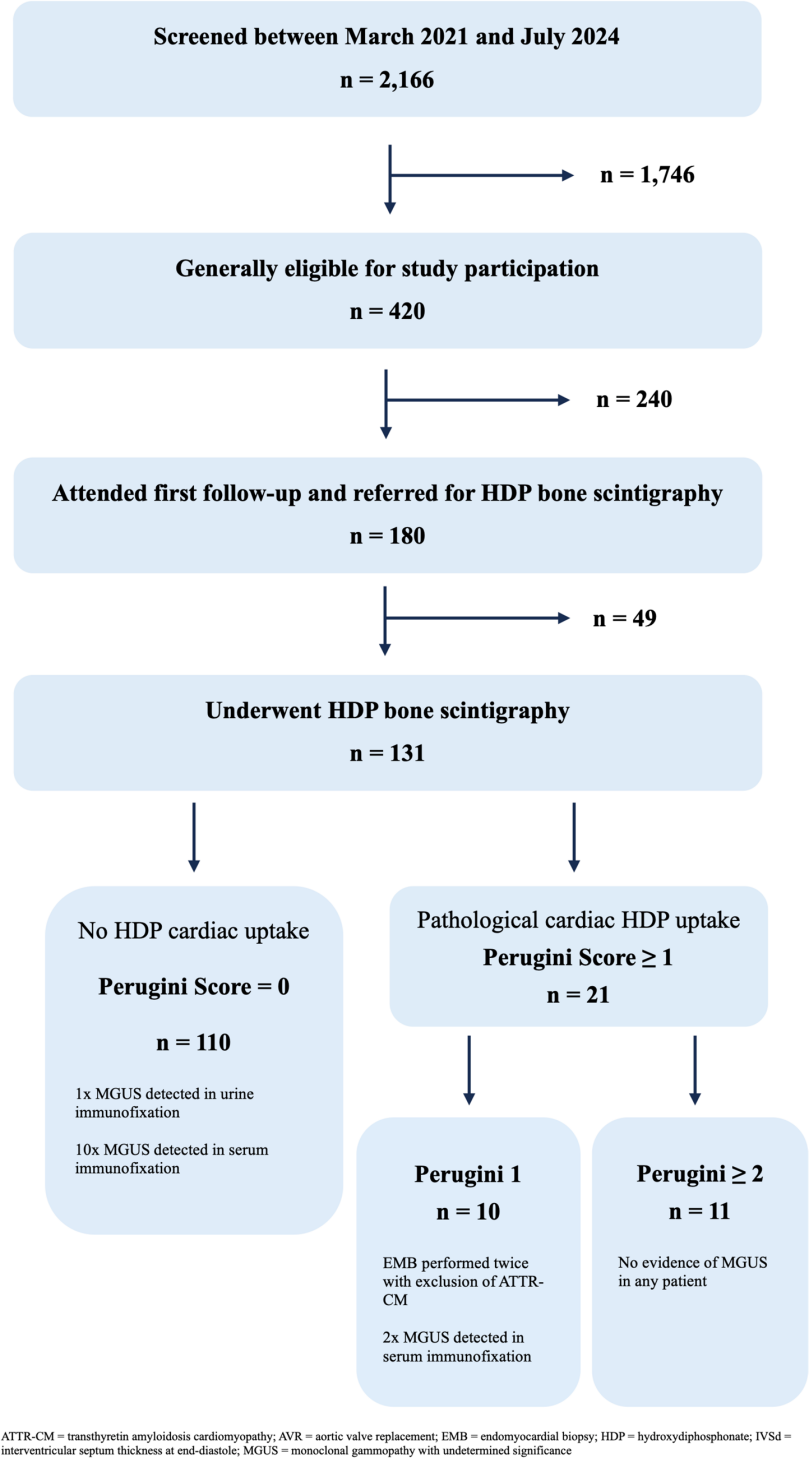
Study flow diagram showing patient enrollment and the results of ATTR-CM diagnostics

Out of 2,166 patients screened, 420 were deemed eligible and scheduled for study enrollment. However, only 180/420 (43%) patients attended the first FU visit and were subsequently planned for bone scintigraphy (Fig. 1). Due to additional dropouts, the final study cohort comprised 131 patients who completed the first clinical FU after AVR and bone scintigraphy. The main reasons for non-attendance were external disruptions (e.g., COVID-19 pandemic, healthcare strikes), patient preference for local care, and clinical factors such as comorbidities, re-hospitalizations, or rehabilitation stays. Most patients underwent TAVR, with only one referred for SAVR (Table 2). Of the total cohort, 105/131 patients (80%) had high-gradient AS, while 26/131 patients (20%) had low-flow, low-gradient AS. The median patient age was 81 years (IQR: 78; 85), and 84/131 patients (64%) were male (Table 3). A total of 20/131 patients (15%) reported a history of CTS.

**Table 2:** Baseline data of the study cohort before AVR

	All patients	Perugini Score 0 (non-pathological bone scintigraphy)	Perugini Score 1–3 (pathological bone scintigraphy)	*p*
	*n* = 131	*n* = 110 (84.0%)	*n* = 21 (16.0%)	
Subtype of AS				
High-gradient; %	80.2	81.8	71.4	
Classical low-flow, low-gradient; %	7.6	6.4	14.3	0.412
Paradoxical low-flow, low-gradient; %	12.2	11.8	14.3	
NYHA class				
I; %	2.3	1.8	4.8	
II; %	30.5	32.7	19.0	0.304
III; %	62.6	60.0	76.2	
IV; %	4.6	5.5	0.0	
Cardiac device; %	12.2	11.8	14.3	0.752
Biomarkers				
NT-proBNP (pg/mL); Mdn [Q_1_; Q_3_]	1,251 [571; 2,477] (*n=*112)	1,058 [485; 2,115] (*n=*94)	2,293 [1,751; 6,396] (*n=*18)	< 0.001
Hs-TnT (ng/mL); Mdn [Q_1_; Q_3_]	0.029 [0.017; 0.043] (*n=*100)	0.024 [0.016; 0.040] (*n=*83)	0.046 [0.029; 0.065] (*n=*17)	0.006
LVEF (%); Mdn [Q_1_; Q_3_]	56 [55; 58]	56 [55; 58]	57 [56; 60]	0.065
Type of procedure				
TAVR; %	99.2	99.1	100.0	0.066
SAVR; %	0.8	0.9	0.0	

*AS *aortic stenosis, *Hs-TnT *high-sensitivity troponin T, *LVEF *left ventricular ejection fraction, *NT-proBNP* N-terminal pro-B-type natriuretic peptide, *NYHA *New York Heart Association, *SAVR *surgical aortic valve replacement, *TAVR *transcatheter aortic valve replacement

**Table 3:** Baseline data of the study cohort at 30-day FU after AVR

	All patients	Perugini Score 0 (non-pathological bone scintigraphy)	Perugini Score 1–3 (pathological bone scintigraphy)	*p*
	*n* = 131	*n* = 110 (84.0%)	*n* = 21 (16.0%)	
Demographic data				
Male; %	64.1	64.5	61.9	0.817
Age (years); Mdn [Q_1_; Q_3_]	81 [78; 85]	81 [77; 84]	85 [83; 87]	< 0.001
BMI (kg/m^2^); Mdn [Q_1_; Q_3_]	26.8 [24.7; 30.7]	26.9 [24.8; 30.9]	25.6 [23.4; 29.2]	0.172
Vital parameters				
SBP (mmHg); M ± SD	148 ± 21 (*n=*129)	149 ± 21 (*n=*108)	146 ± 21	0.508
DBP (mmHg); M ± SD	74 ± 10 (*n=*129)	74 ± 10 (*n=*108)	74 ± 9	0.946
Heart rate (1/min); Mdn [Q_1_; Q_3_]	72 [63; 80]	72 [63; 81]	72 [64; 78]	0.918
Symptoms				
NYHA class				
I; %	17.6	16.4	23.8	
II; %	68.7	70.0	61.9	0.814
III; %	13.0	12.7	14.3	
IV;%	0.8	0.9	0.0	
Decline in NYHA class ≥ I; %	67.2	65.5	76.2	0.337
Comorbidities				
Arterial hypertension; %	89.3	89.1	90.5	0.851
CAD; %	52.7	52.7	52.4	0.977
History of myocardial infarction; %	4.6	5.5	0.0	0.273
AF; %	38.9	34.5	61.9	0.018
Previous cardiothoracic surgery; %	6.9	8.2	0.0	0.174
CKD with dialysis; %	1.5	1.8	0.0	0.533
Cardiac device therapy; %	16.0	15.5	19.0	0.681
Potential extracardiac manifestations of ATTR				
History of CTS; %	15.3	11.8	33.3	0.012
Lumbar spinal stenosis; %	9.2	9.1	9.5	0.950
Clinical signs of PNP; %	22.9	24.5	14.3	0.305
Biomarkers				
Hemoglobin (g/dL); M ± SD	12.4 ± 1.8	12.4 ± 1.8	12.6±1.8	0.621
Creatinine (mg/dL); Mdn [Q_1_; Q_3_]	1.10 [0.80; 1.40]	1.00 [0.80; 1.33]	1.50 [1.00; 1.75]	0.008
GFR (mL/min); Mdn [Q_1_; Q_3_]	63 [44; 77]	66 [47; 80]	42 [32; 70]	0.003
NT-proBNP (pg/mL); Mdn [Q_1_; Q_3_]	727 [303; 1,458]	619 [254; 1,352]	1,519 [909; 2,386]	< 0.001
Δ NT-proBNP decline (pg/mL); Mdn [Q_1_; Q_3_]	322 [0; 1119] (*n=*112)	257 [–35; 1018] (*n=*94)	686 [104; 2777] (*n=*18)	0.085
Hs-TnT (ng/mL); Mdn [Q_1_; Q_3_]	0.025 [0.016; 0.041]	0.023 [0.015; 0.038]	0.037 [0.024; 0.055]	0.005
Δ hs-TnT decline (ng/mL); Mdn [Q_1_; Q_3_]	0.000 [–0.006; 0.007] (*n=*100)	0.001 [–0.006; 0.007] (*n=*83)	0.004 [–0.005; 0.016] (*n=*17)	0.363
Electrocardiogram				
Rhythm				
Sinus; %	71.8	70.9	76.2	0.791
AF*; %	19.8	20.0	19.0	
VAT mode or atrial paced; %	8.4	9.1	4.8	
Atrioventricular conduction				
Normal PQ interval; %	53.4	54.5	47.6	0.413
Atrioventricular block I°; %	18.3	16.4	28.6	
AF or paced; %	28.2	29.1	23.8	
QRS width (ms); Mdn [Q_1_; Q_3_]	100 [90; 140]	100 [90; 140]	106 [90; 140]	0.927
QRS configuration				
Normal; %	55.0	54.5	57.1	0.960
LAHB; %	4.6	4.5	4.8	
LBBB; %	17.6	18.2	14.3	
RBBB; %	12.2	12.7	9.5	
Pacing*; %	10.7	10.0	14.3	
QRS-Score (mV)**; M ± SD	13.46 ± 3.31 (*n=*78)	13.63 ± 3.27 (*n=*65)	12.58 ± 3.49 (*n=*13)	0.383
Sokolow-Lyon-Index (mV)**; Mdn [Q_1_; Q_3_]	2.40 [1.78; 3.00] (*n=*78)	2.40 [1.80; 2.95] (*n=*65)	2.20 [1.65; 3.05] (*n=*13)	0.747
Low voltage; %	6.1	5.5	9.5	0.475
Imaging				
IVSd (mm); Mdn [Q_1_; Q_3_]	14 [13; 15]	14 [13; 15]	15 [13; 18]	0.033
LVEDD (mm); M ± SD	47 ± 7	47 ± 8	46 ± 6	0.867
PWT (mm); Mdn [Q_1_; Q_3_]	12 [10; 13]	12 [10; 13]	12 [9; 15]	0.621
RV free wall thickness (mm); Mdn [Q_1_; Q_3_]	7 [6; 8] (*n=*124)	7 [6; 8] (*n=*103)	8 [7; 9]	0.072
LVMI (g/m^2^); Mdn [Q_1_; Q_3_]	116 [98; 141] (*n=*128)	116 [98; 139] (*n=*107)	127 [100; 159]	0.403
LVEF (%); Mdn [Q_1_; Q_3_]	57 [55; 59]	57 [55; 58]	58 [55; 61]	0.188
GLS (%)**; M ± SD	–16.4 ± 4.5 (*n=*73)	–16.4 ± 4.6 (*n=*59)	–16.2 ± 4.2 (*n=*13)	0.903
Apical sparing**; %	16.4 (*n=*73)	10.0 (*n=*60)	46.2 (*n=*13)	0.001
Apical sparing index**; Mdn [Q_1_; Q_3_]	0.80 [0.64; 0.96] (*n=*47)	0.77 [0.58; 0.91] (*n=*36)	0.88 [0.82; 1.40] (*n=*11)	0.009
LAVI (mL/m^2^); Mdn [Q_1_; Q_3_]	41 [34; 54]	42 [34; 54]	40 [35; 57]	0.903
SVI (mL/m^2^); Mdn [Q_1_; Q_3_]	41 [35; 48] (*n=*127)	41 [36; 48] (*n=*106)	41 [30; 46]	0.277
FAC (%); Mdn [Q_1_; Q_3_]	45 [40; 49] (*n=*126)	45 [40; 49] (*n=*106)	45 [40; 49] (*n=*20)	0.794
TAPSE (mm); Mdn [Q_1_; Q_3_]	21 [19; 24]	21 [19; 25]	22 [19; 24]	0.753
RA area (cm^2^); Mdn [Q_1_; Q_3_]	17 [14; 22]	17 [14; 22]	19 [16; 26]	0.168
RVEDD (mm); Mdn [Q_1_; Q_3_]	36 [32; 39]	36 [31; 39]	37 [33; 39]	0.701
AV v_max_ (m/s); M ± SD	2.08 ± 0.46	2.08 ± 0.45	2.07 ± 0.54	0.879
AV dP_max_ (mmHg); Mdn [Q_1_; Q_3_]	18 [13; 23]	18 [13; 23]	15 [11; 25]	0.598
AV dP_mean_ (mmHg); Mdn [Q_1_; Q_3_]	9 [7; 12]	9 [7; 12]	9 [6; 14]	0.809
AVA (cm^2^); Mdn [Q_1_; Q_3_]	1.90 [1.60; 2.28] (*n=*128)	1.95 [1.60; 2.30] (*n=*107)	1.70 [1.57; 2.10]	0.154
PVL ≥ moderate; %	7.6	7.3	9.5	0.426
E/A; Mdn [Q_1_; Q_3_]	0.75 [0.60; 1.00] (*n=*104)	0.72 [0.58; 0.96] (*n=*87)	0.88 [0.77; 1.60] (*n=*17)	0.007
E _max_ (cm/s); Mdn [Q_1_; Q_3_]	87 [65; 115] (*n=*124)	84 [64; 115] (*n=*103)	91 [73; 122]	0.246
A _max_ (cm/s); M ± SD	107 ± 32 (*n=*104)	109 ± 31 (*n=*87)	95 ± 38 (*n=*17)	0.112
DT (ms); M ± SD	250 ± 89 (*n=*122)	247 ± 86 (*n=*101)	266 ± 102	0.373
Septal e‘ (cm/s); Mdn [Q_1_; Q_3_]	6 [5; 7] (*n=*119)	6 [5; 7] (*n=*99)	6 [4; 6] (*n=*20)	0.395
Lateral e‘ (cm/s); Mdn [Q_1_; Q_3_]	8 [6; 10] (*n=*119)	8 [6; 10] (*n=*99)	7 [6; 10] (*n=*20)	0.631
Mitral s‘ (cm/s); Mdn [Q_1_; Q_3_]	7 [6; 9] (*n=*117)	7 [7; 9] (*n=*97)	7 [6; 8] (*n=*20)	0.018
E/e’; Mdn [Q_1_; Q_3_]	12.7 [9.9; 17.0] (*n=*117)	12.7 [9.8; 16.1] (*n=*97)	14.1 [9.9; 20.0] (*n=*20)	0.348
TR v_max_ (m/s); Mdn [Q_1_; Q_3_]	2.7 [2.4; 2.9] (*n=*118)	2.7 [2.3; 2.9] (*n=*97)	2.8 [2.6; 3.0]	0.156
TR dP_max_ (mmHg); Mdn [Q_1_; Q_3_]	29.0 [22.8; 35.0] (*n=*118)	28.0 [21.6; 35.0] (*n=*97)	30.0 [26.0; 36.0]	0.141
sPAP (mmHg); Mdn [Q_1_; Q_3_]	33.0 [26.0; 39.3] (*n=*118)	32.0 [25.0; 39.0] (*n=*97)	35.0 [30.0; 40.0]	0.169
Severe MS; %	0.0	0.0	0.0	n.a.
Severe MR; %	0.0	0.0	0.0	n.a.
Severe TR; %	3.1	3.7	0.0	0.373
Pericardial effusion; %	3.1	3.6	0.0	0.375
Scores				
RAISE-Score ≥ 2; %	48.9	43.6	76.2	0.006
RAISE Score ≥ 3; %	28.2	21.8	61.9	< 0.001
H2FPEF Score ≥ 5; %	80.9	80.0	85.7	0.541
Amyloidosis diagnostics				
HDP bone scintigraphy				
Perugini 0; %	84.0	100.0	0.0	
Perugini 1; %	7.6	0.0	47.6	< 0.001
Perugini 2–3; %	8.4	.0	52.4	
Serum immunofixation				
No MGUS; %	74.8	72.7	85.7	
MGUS; %	9.2	9.1	9.5	0.486
Inconclusive; %	13.7	15.5	4.8	
Not done; %	2.3	2.7	0.0	
EMB; %	1.5	0.0	9.5	0.001
Normal finding; %	1.5	0.0	100.0	n.a.
Pathological finding; %	0.0	0.0	0.0	n.a.
ATTR-CM				
None; %	85.5	100.0	9.5	
ATTR-CM; %	8.4	0.0	52.4	< 0.001
Not classifiable due to missing EMB; %	6.1	0.0	38.1	
ATTR specific medication				
Tafamidis 61mg; %	8.4	0.0	52.4	< 0.001

*4 patients with atrial fibrillation and ventricular pacing; **only patients without ventricular pacing and complete bundle branch block; *AF *atrial fibrillation, *ATTR *transthyretin amyloidosis, *ATTR-CM *transthyretin amyloidosis cardiomyopathy, *AV *aortic valve, *AVA *aortic valve area, *BMI *body mass index, *CAD *coronary artery disease, *CKD *chronic kidney disease, *CTS* carpal tunnel syndrome, *DBP *diastolic blood pressure, *dP *differential pressure, *DT *deceleration time, *EMB *endomyocardial biopsy, *FAC *fractional area change, *GFR* glomerular filtration rate, *GLS *global longitudinal strain, *HDP *hydroxydiphosphonate, *Hs-TnT *high-sensitive troponin T, *IVSd* interventricular septum thickness at end-diastole, *LAHB* left anterior hemiblock, *LAVI *left atrial volume index, *LBBB *left bundle branch block, *LVEDD *left ventricular end-diastolic diameter, *LVEF *left ventricular ejection fraction, *LVMI *left ventricular mass index, *M *mean, *MAPSE *mitral annular plane systolic excursion, *Mdn *median, *MGUS *monoclonal gammopathy with undetermined significance, *MR *mitral regurgitation, *MS *mitral stenosis, *n.a*. not available, *NT-proBNP *N-terminal pro-B-type natriuretic peptide, *NYHA *New York Heart Association, *PNP *polyneuropathy, *PVL *paravalvular leak, *PWT* posterior wall thickness, *Q *quartile, *RA *right atrium, *RBBB *right bundle branch block, *RV *right ventricular, *RVEDD *right ventricular end-diastolic diameter, *SBP *systolic blood pressure, *SD *standard deviation, *sPAP *systolic pulmonary arterial pressure, *SVI *stroke volume index, *TAPSE *tricuspid annular plane systolic excursion, *TR *tricuspid regurgitation, *v *velocity, *VAT mode* pacemaker senses electrical signals from the atrium and stimulates the ventricle to maintain synchrony between the atrium and ventricle

Before AVR, 11/131 patients (8%) had a left bundle branch block (LBBB), 20/131 (15%) had a RBBB, and 6/131 (5%) showed ventricular pacing on ECG. Dyspnea classified as New York Heart Association (NYHA) class III or IV was present in 88/131 patients (67%). The median N-terminal pro-B-type natriuretic peptide (NT-proBNP) and hs-TnT levels before AVR were 1,251 pg/mL (IQR: 571; 2477) and 0.029 ng/mL (IQR: 0.017; 0.043), respectively.

At the 30-day FU after AVR, 94/131 patients (72%) were in sinus rhythm. Twenty-three of 131 patients (18%) had a LBBB, 16/131 (12%) had a RBBB, and 14/131 (11%) showed a ventricular paced rhythm. In patients without pacing and without complete bundle branch block (BBB), the median Sokolow-Lyon-Index was 2.40 mV (IQR: 1.78; 3.00). Improvement in dyspnea was observed in approximately two-thirds of the patients but 18/131 patients (14%) remained in NYHA class III or higher. The median cardiac biomarker levels were 727 pg/mL (IQR: 303; 1,458) for NT-proBNP and 0.025 ng/mL (IQR: 0.016; 0.041) for hs-TnT. There was a significant decrease in NT-proBNP levels (p < 0.001) following AVR, whereas hs-TnT levels (p = 0.512) did not show a relevant decline. The median IVSd was 14 mm (IQR: 13; 15), and the left ventricular ejection fraction (LVEF) was 57% (IQR: 55; 59). Among patients for whom it was measurable, the mean global longitudinal strain (GLS) was −16.4 ± 4.5%. The median E/A ratio was 0.75 (IQR: 0.60; 1.00).

A RAISE Score of ≥ 2 and ≥ 3 was found in 64/131 patients (49%) and 37/131 patients (28%), respectively. Pathological bone scintigraphy results were observed in 21/131 patients (16%). Of these, ten had a Perugini Score of 1, and eleven patients had a Perugini Score of ≥ 2 (Tables S1-S3). MGUS was not found in any patient with a Perugini Score ≥ 2 and hence all these were diagnosed with ATTR-CM (11/131; 8%). For patients with a Perugini Score of 1 on bone scintigraphy, an EMB was recommended, but only two patients agreed to it, both of whom showed no evidence of amyloid. In total, ATTR-CM was diagnosed in eleven patients, while eight patients remained with an unclear diagnosis due to the lack of EMB. All patients diagnosed with ATTR-CM were subsequently treated with the TTR stabilizer tafamidis.

Median FU of the study cohort was 19 months (IQR: 9; 35). Information on all-cause mortality and HF hospitalization was missing for two and six patients, respectively.

### Performance of the RAISE Score

The sensitivity and specificity of a RAISE Score ≥ 2 for identifying patients with pathological bone scintigraphy (Perugini 1–3) were 76% and 56%, respectively, with a PPV of 25% and a NPV of 93% (Table 4A). Using a RAISE Score ≥ 3 as the cutoff reduced sensitivity to 62% but increased specificity to 78%. The PPV and NPV were 35% and 92%, respectively (Table 4C).

**Table 4:**
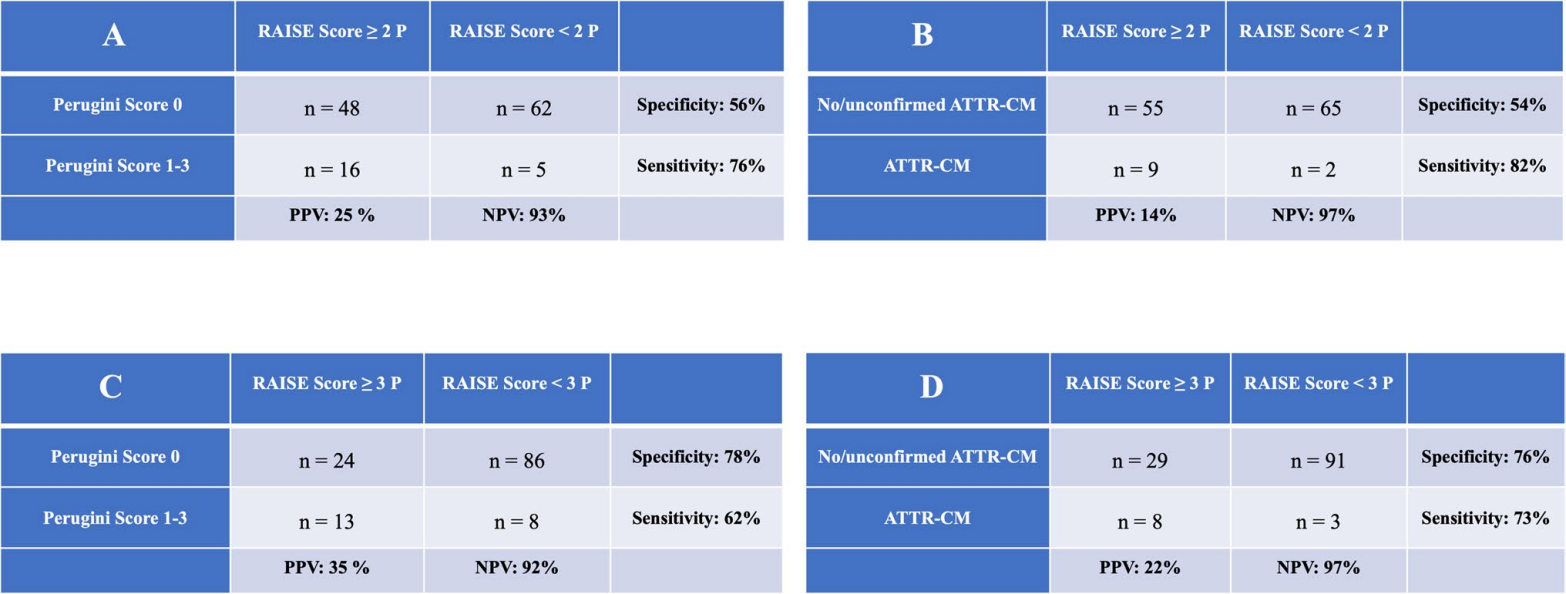
Sensitivity, specificity, PPV, and NPV for the RAISE Score with cutoff values of 2 (**A** and **B**) and 3 (**C** and **D**) regarding pathological bone scintigraphy result and definitive ATTR-CM diagnosis

For the diagnosis of definitive ATTR-CM, the RAISE Score had a sensitivity of 82% and a specificity of 54% at a cutoff of ≥ 2 (Table 4B). With a cutoff of ≥ 3, sensitivity decreased to 73%, while specificity increased to 76% (Table 4D). The PPV for these cutoffs were 14% and 22%, respectively, and the NPV remained 97% for both.

**Fig. 2:**
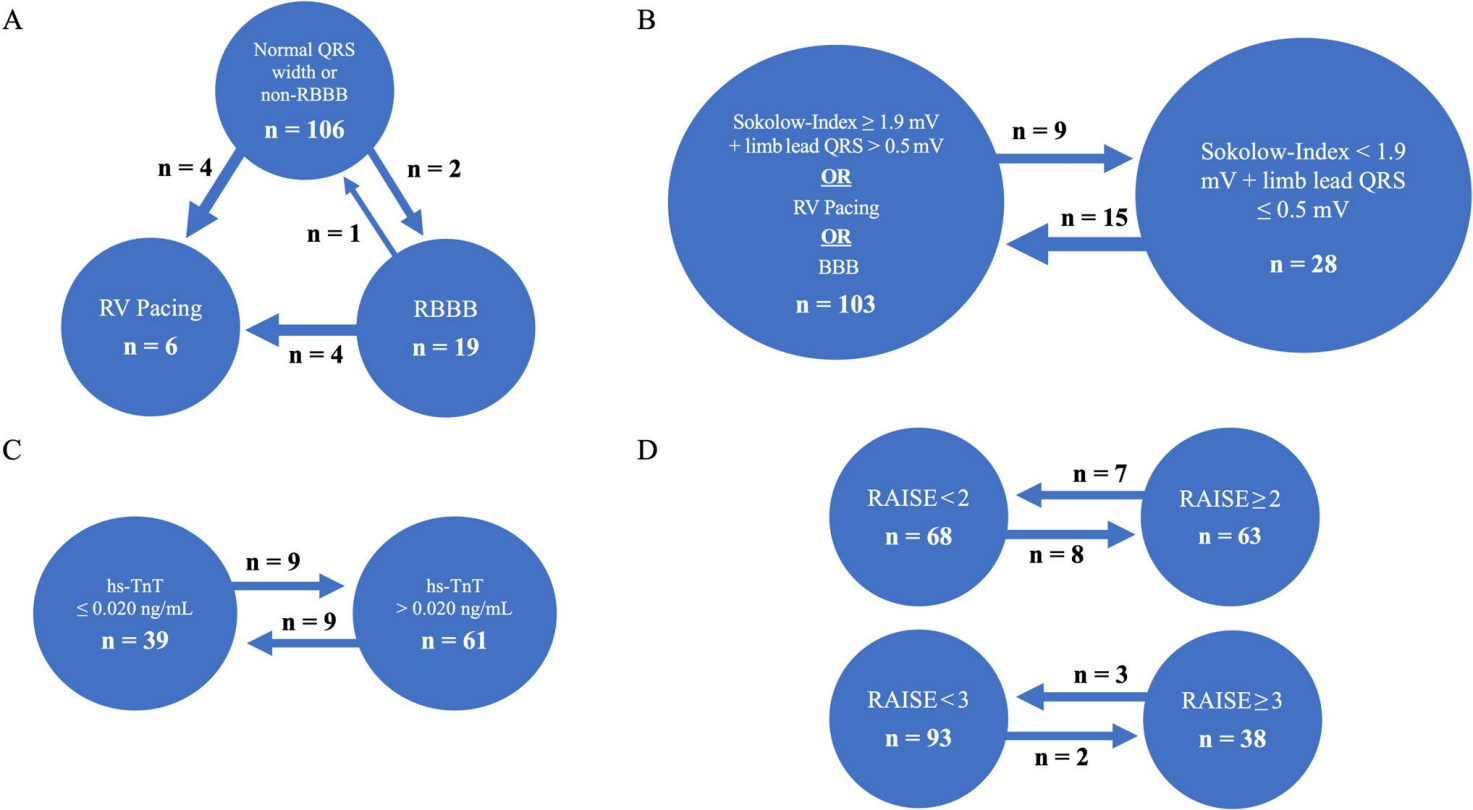
Pre-procedural classifications for variables potentially influenced by AVR are depicted within the circles. Arrows indicate patients whose classification changed following AVR. RBBB criterion of the RAISE Score (**A**). Low-voltage criterion of the RAISE Score (**B**). Hs-TnT criterion of the RAISE Score (pre-procedural data available for 100 patients) (**C**). Final RAISE Score classification (**D**). With respect to changes in the RAISE Score related to threshold 2, only two patients with a pathological bone scintigraphy were identified—both had a RAISE Score ≥ 2 after AVR. Regarding changes in the RAISE Score related to threshold 3, no patient had a pathological bone scintigraphy. For the 31 patients with missing pre-procedural hs-TnT levels, post-procedural values were used for both calculations

With respect to RAISE Score items that could potentially have been influenced by AVR, changes in pre- and post-procedural classifications are presented in Fig. 2 Overall, 43 patients showed changes in ECG criteria and/or hs-TnT levels after AVR, though these changes did not always lead to discordant RAISE Score classification according to the proposed thresholds. Using pre-procedural ECG or hs-TnT levels would not have changed diagnostic accuracy, with RAISE ≥ 2 yielding 67% sensitivity and 55% specificity and RAISE ≥ 3 yielding 62% and 77%, respectively.

### Additional pre-selection criteria

In terms of clinical characteristics, patients with pathological findings on bone scintigraphy were significantly older and more frequently diagnosed with atrial fibrillation (AF) (62% vs. 35%; p = 0.018) and CTS (33% vs. 12%; p = 0.012) compared to those with normal results (Table 3). Additionally, patients with findings suggestive of ATTR-CM exhibited markedly elevated levels of post-procedural NT-proBNP (1,519 vs. 619 pg/mL; p < 0.001) and hs-TnT (0.037 vs. 0.023 ng/mL, p = 0.005). There was no significant difference in the decline of cardiac biomarkers between patients with and without pathological bone scintigraphy. Kidney function was also significantly more impaired in patients with pathological bone scintigraphy, as indicated by higher creatinine levels and reduced glomerular filtration rate (GFR). In this subgroup, TTE measurements showed higher IVSd and E/A ratios, while mitral s’ velocity was lower. Additionally, apical sparing was more frequently observed. ROC analyses regarding pathological bone scintigraphy were conducted for age, NT-proBNP, hs-TnT, GFR, IVSd, E/A ratio, and mitral s’ (Figs. S1-7; Table S4).

Among all variables, age exhibited the highest area under the curve (AUC), with a discriminative threshold of 82.6 years. For biomarkers, NT-proBNP showed the best AUC, with a threshold value of 1,433 pg/mL. For identifying patients with pathological bone scintigraphy, an age threshold of ≥ 83 years achieved a sensitivity of 81%, a specificity of 68%, a PPV of 33%, and a NPV of 95% (Table 5A). For the diagnosis of ATTR-CM, corresponding values are shown in Table 5B. The combination of the clinical variables CTS and/or NT-proBNP level ≥ 1,400 pg/mL yielded a sensitivity of 81%, a specificity of 66%, a PPV of 31%, and a NPV of 95% for identifying patients with pathological bone scintigraphy (Table 5C). This combination outperformed NT-proBNP ≥ 1,400 pg/mL alone (Table S5). For the diagnosis of ATTR-CM, this approach achieved a sensitivity of 91%, a specificity of 63%, a PPV of 19%, and a NPV of 99% (Table 5D). Non-inferiority analysis was positive for age ≥ 83 years and CTS and/or NT-proBNP level ≥ 1,400 pg/mL compared to a RAISE Score ≥ 3, but not compared to RAISE Score ≥ 2.

**Table 5:**
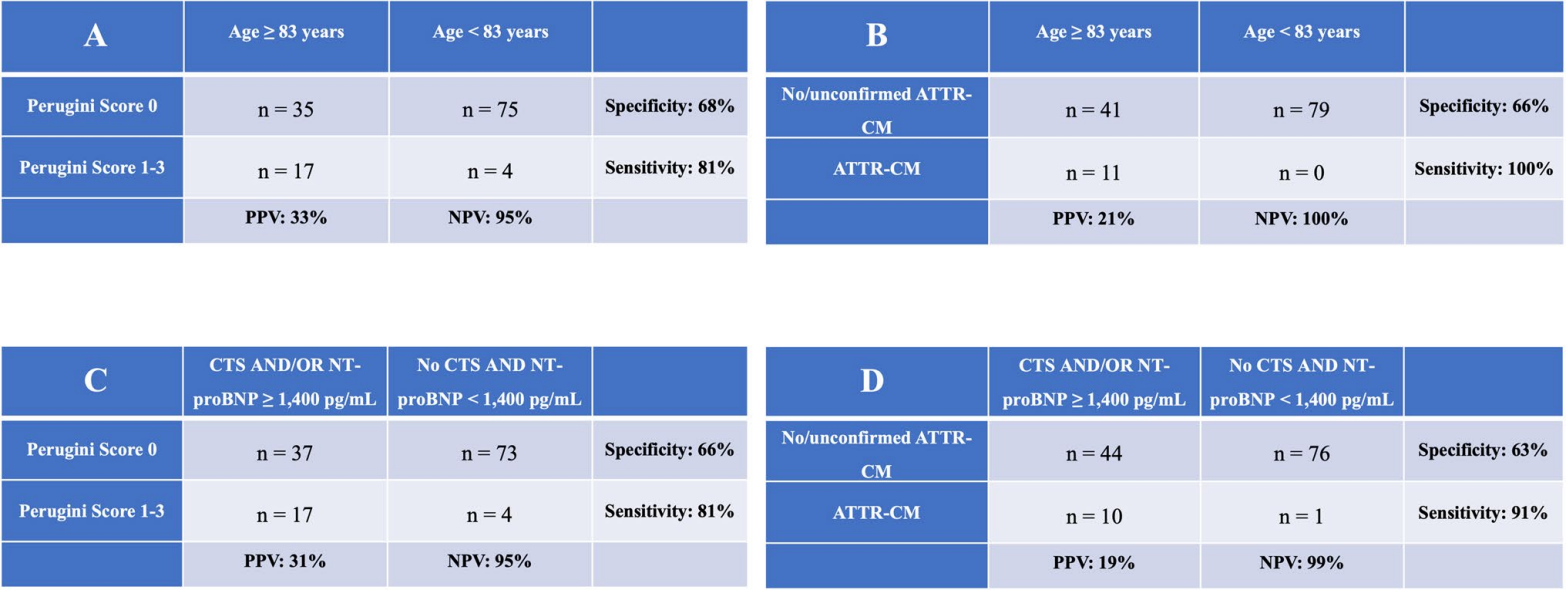
Sensitivity, specificity, PPV, and NPV for age ≥ 83 years (**A** and **B**) and the combination of CTS and/or NT-proBNP ≥ 1,400 pg/mL (**C** and **D**) regarding pathological bone scintigraphy result and definitive ATTR-CM diagnosis

### Clinical outcome

**Fig. 3:**
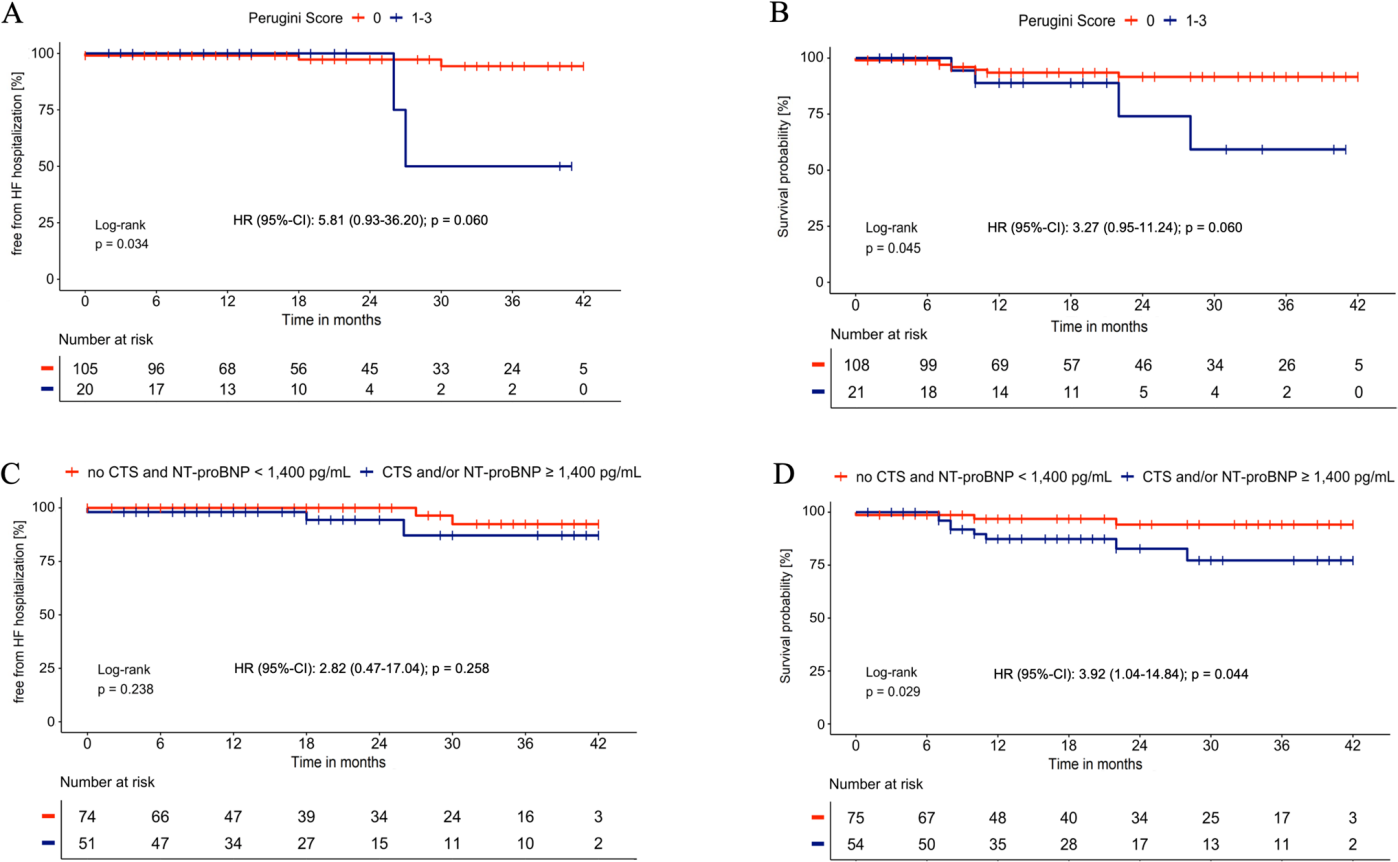
Kaplan–Meier curves regarding HF hospitalization (**A** and **C**) and all-cause mortality (**B** and **D**) based on the results of bone scintigraphy and presence of CTS and/or NT-proBNP ≥ 1,400 pg/mL

The overall survival rate was 89% at 2 years (Fig. S8). Patients with pathological bone scintigraphy results had higher event rates for hospitalization due to HF (50% vs. 5.7% at 3-years; Fig. 3A) and higher all-cause mortality compared to those without cardiac HDP uptake (2-year mortality: 26% vs. 8%; Fig. 3B). Although the Log-Rank test was significant for both endpoints, the p-value from the Cox regression narrowly missed statistical significance in both analyses.

All-cause mortality did not differ significantly between patients with or without a RAISE Score of ≥ 2 (p = 0.750) or ≥ 3 (p = 0.519) (Figs. S9 and S10). The same applied to patients aged ≥ 83 years (p = 0.346) (Fig. S11). Regarding HF hospitalization, no significant differences were observed for a RAISE Score ≥ 2 (p = 0.147), whereas a RAISE Score ≥ 3 and an age ≥ 83 years were associated with higher rates of cardiac decompensation (p = 0.005 and p = 0.047) (Figs. S12-14).

The combination of CTS and/or NT-proBNP level ≥ 1,400 pg/mL was significantly associated with all-cause mortality (p = 0.029; Fig. 3D), but not with HF hospitalization rate (p = 0.238; Fig. 3C).

## Discussion

The main findings of this study are:

i) Pathological bone scintigraphy results (Perugini Score ≥ 1) were found in 16% of the cohort, but only half of these patients had a Perugini Score ≥ 2 and a confirmed diagnosis of ATTR-CM.
ii) The diagnostic accuracy of the RAISE Score thresholds following AVR remained limited, with high NPV but low PPV and sensitivity.
iii) Age, CTS history, and NT-proBNP levels emerged as the most valuable and objective parameters for ATTR-CM screening in AS patients.
iv) All-cause mortality trended toward association with pathological bone scintigraphy and was significantly associated with CTS history and/or NT-proBNP level ≥ 1,400 pg/mL, whereas the RAISE Score was not.

### Prevalence of pathological bone scintigraphy and ATTR-CM

In our predominantly male cohort of octogenarians, almost all treated with TAVR, 16% showed pathological findings on bone scintigraphy. Reported prevalences of ATTR-CM in patients with severe AS range from 4 to 16% [10, 11, 20–22]. However, many studies lacked precise definitions of ATTR-CM, interpreting a Perugini Score of 1 as diagnostic for ATTR-CM [11, 21, 23]. Scully et al. found that 13% of 200 TAVR patients had ATTR-CM, but nearly one-third had only a Perugini Score of 1 in bone scintigraphy [23]. Similarly, Nitsche et al. reported ATTR-CM in 11.9% of 407 AS patients, yet only 32 had a Perugini Score ≥ 2, and only 37 underwent AVR [11]. This suggests that the actual rate of confirmed ATTR-CM in these studies is below 10%, which aligns with our findings of 8.4%.

However, it must be noted that most patients with an indication for EMB declined the procedure, and potential selection bias may have led to underrepresentation of patients with poorer health status who are more likely to have ATTR-CM. In contrast, Castano et al. appropriately applied a Perugini Score ≥ 2 to diagnose ATTR-CM and reported a higher prevalence of 16% [10]. In summary, ATTR-CM might affect approximately one in ten patients with AS—rather than one in seven – thus moderating the strength of this association.

A Perugini Score of 1 was also common in our cohort. According to ESC Guidelines, this result necessitates a diagnostic EMB, a more invasive and time-intensive procedure [16]. Many octogenarians decline invasive diagnostics, particularly shortly after recovering from AVR. Performing bone scintigraphy prior to AVR could enable EMB during AVR, but this approach presents several challenges: First, outpatient bone scintigraphy in close relation to AVR is logistically difficult to implement in frail and elderly patients and is not reimbursed as part of the AVR procedure; Second, both AVR and EMB carry independent risks of complications, including disabling strokes, pericardial tamponade or major bleeding and concomitant biopsy will prolong the overall procedural time [15]. Third, given the unclear clinical and functional improvement in patients with severe AS and ATTR-CM, there may be no indication for specific ATTR-CM treatment after AVR due to the clinical status, thus making an EMB unnecessary. Therefore, a stepwise approach—starting with AVR, followed by ATTR-CM evaluation and potential treatment—seems pragmatic and aligns with the conclusions of our study.

### Performance of the RAISE Score

The RAISE Score, developed by Nitsche et al., has not been prospectively validated in an external cohort where all patients underwent bone scintigraphy, regardless of their initial scoring [11]. To date, additional analyses of the RAISE Score rely solely on retrospective data, case reports, or studies where scintigraphies were only performed in patients pre-selected clinically or based on the Score [24–27]. Nitsche et al. reported sensitivities of 94% for a Score of ≥ 2 and 72% for a Score of ≥ 3 [11]. In our cohort, sensitivity was lower at 76% and 62%, respectively – representing a performance drop of 10% to 18%. While the RAISE Score demonstrates moderate to good sensitivity as a screening tool for ATTR-CM, its specificity in our study aligned with Nitsche et al.’s findings [11]. The PPV ranged from 25 to 35%, while the NPV consistently exceeded 90% across all cutoffs.

The high NPV underscores the clinical utility of the RAISE Score by enabling reliable preselection and minimizing unnecessary further testing, thereby supporting cost-effective and resource-efficient management—particularly in patients with AS [28]. If a RAISE Score ≥ 2 had been used as a screening tool prior to bone scintigraphy, 64 scans instead of the full 131 would have been performed. Assuming a cost of ~ €470 per scan, €31,490 would have been saved, at the cost of missing 24% (5/21) of patients with actual pathological scan results [29]. While the cost of TTR stabilizer treatment far exceeds diagnostic expenses, the high organizational effort and inefficient use of nuclear medicine resources make pre-selection essential. A screening tool must be practical and capable of detecting ATTR-CM at an early disease stage to allow for treatment options [2, 3, 16]. However, the RAISE Score includes an IVSd ≥ 18 mm and age ≥ 85 years, both of which indicate advanced disease stages, which could be criticized [11]. The goal should not simply be to detect ATTR-CM but to identify patients at a stage where treatment would be most beneficial.

### Most valuable pre-selection criteria

Five of the seven RAISE Score parameters also predicted pathological bone scintigraphy in our cohort, except for the two ECG variables. Although AVR can lead to pacemaker implantation, new BBB or change of QRS axis and amplitude, the use of the pre-procedural ECGs would not have changed our results significantly [15, 30]. However, the ROC-derived thresholds differed slightly from the original publication and were calculated based on patients diagnosed with definitive ATTR-CM [11]. The best screening parameter, based on AUC, was age ≥ 83 years, which was comparable to the AUC for the full RAISE Score reported by Nitsche et al. [11]. All patients with diagnoses of ATTR-CM in this cohort were ≥ 83 years, consistent with other studies that show ATTR-CM detection primarily in octogenarians with AS [11, 20, 23].

The association between AS and ATTR-CM remains debated, with some hypotheses suggesting that AS either results from amyloidosis or promotes amyloid deposition due to increased wall stress [31, 32]. However, given that both conditions are strongly age-related, an epidemiological coincidence seems more likely than a direct causal link. Amyloid deposits were found in 25% of patients over 85 years in an autopsy study, and relevant cardiac tracer uptake (Perugini Score ≥ 2) was detected in 13.9% of males without any clinical suspicion of ATTR-CM [33, 34]. Although these findings do not necessarily confirm manifest ATTR-CM, as specific imaging criteria must be met for diagnosis, they highlight the increased likelihood of amyloid detection with age [2]. Interestingly, Chacko et al. found no increased prevalence of severe AS in 766 patients with wild-type ATTR-CM, with a mean age of 78 years [35]. In this context, the RAISE Score or the proposed pre-selection criteria may be useful not only as a screening tool for ATTR-CM in patients with AS but also as a general screening tool.

While we acknowledge an association between AS and ATTR-CM to some extent, with advancing age this relationship may become increasingly coincidental, suggesting that age has limited value as a screening criterion and raising concerns regarding false-positive findings. Moreover, the fact that most ATTR-CM patients screened due to AS were ≥ 85 years raises doubts about the overall value of this approach. Focusing on early detection and cardiac biomarkers, we found that NT-proBNP was a better discriminator than hs-TnT in our cohort, with a cutoff value of ≥ 1,400 pg/mL. This threshold is lower than the 3,000 pg/mL threshold used in the National Amyloidosis Centre (NAC) staging system but still enables diagnosis in stage I [36]. Combined with CTS, a commonly recognized early red flag for ATTR-CM, this approach performed similarly to age ≥ 83 years and was non-inferior to the RAISE Score ≥ 3. Nevertheless, both NT-proBNP and CTS have their limitations. NT-proBNP is not specific to ATTR-CM and may be elevated in other cardiac conditions or chronic kidney disease [37]. Although amyloid deposits have been found in up to 34% of patients with CTS, the presence of CTS is likely a less reliable predictor of manifest ATTR-CM in younger patients [38]. Therefore, this approach requires validation in a less elderly cohort.

### Clinical outcome

Besides the primary aim of evaluating the performance of the RAISE Score and other pre-selection criteria in relation to bone scintigraphy results, an additional prognostic value of screening tools with respect to all-cause mortality and HF-related hospitalization could trigger closer FU for these higher risk patients. The 2-year all-cause mortality rate was 11%, which is comparable to the rate reported for intermediate-risk patients undergoing TAVR in the PARTNER 2A trial [39]. Although NYHA class after AVR improved to a similar extent in both subgroups, patients with pathological bone scintigraphy had more than three times the 2-year all-cause mortality compared to those with normal scans, according to Kaplan–Meier analysis (26% vs. 8%). Nitsche et al. observed a similar 2-year all-cause mortality for their ATTR-CM subgroup after TAVR but reported a notably high ~ 20% mortality rate in patients with normal scintigraphy results [11]. While they observed significantly better survival with AVR compared to conservative AS treatment, no survival difference was noted between AVR patients with and without ATTR-CM, which stands in contrast to our observations [11]. However, their high all-cause mortality in patients with isolated AS and an intermediate operative risk (mean EuroSCORE II of 4.2) remains unexplained, despite their slightly older age and the fact that AVR was performed between 2016 and 2019 [11]. Recently, we reported that in a real-world ATTR-CM cohort primarily treated with tafamidis, 1-year survival exceeded 95% [7]. Despite the challenges in comparing these cohorts, we hypothesize that patients with AS undergoing AVR and pathological bone scintigraphy scans have a worse prognosis than those with either condition alone.

Since conservative treatment for symptomatic AS results in over 50% mortality within two years, TAVR should not be withheld, even in the presence of ATTR-CM [40]. The RAISE Score itself did not provide prognostic value in this cohort except for RAISE Score ≥ 3 in relation to HF hospitalization. However, it was not specifically designed for this purpose [11]. The combination of a history of CTS and/or NT-proBNP level ≥ 1,400 pg/mL was associated with higher mortality, which is consistent with evidence from a meta-analysis demonstrating higher midterm mortality in patients with elevated NT-proBNP levels following TAVR [41]. These patients should not only undergo further ATTR-CM diagnostics but also be closely monitored even in the case of a negative scan result.

## Study Limitations

This study was initially designed to recruit a much larger number of patients. However, dropout rates were high due to multiple factors, including the COVID-19 pandemic and patients´ reluctance to undergo further diagnostics shortly after AVR. This may have introduced selection bias favoring healthier patients who are less likely to have ATTR-CM. The low willingness among patients with Perugini Score 1 to undergo EMB, limited the detection of additional positive cases, which could have influenced the diagnostic performance of the RAISE Score and other variables. Nevertheless, no relevant differences were observed compared to the overall TAVR cohort at our center, supporting the assumption that the study population is representative [42]. Although our cohort was relatively small, it still comprised approximately two-thirds of the cohort size used by Nitsche et al. to derive the RAISE Score [11].

In contrast to the original RAISE Score, we used ECG and hs-TnT data collected after AVR for our analyses. With respect to hs-TnT, levels remained higher in patients with pathological HDP uptake even after AVR, and the decrease in hs-TnT levels was not significantly different between those with and without pathological scan results. However, pre-procedural hs-TnT levels were unavailable in 31 patients, and AVR may have contributed to deviating hs-TnT levels, which could have influenced RAISE Score classification in 15 of these patients in relation to the defined thresholds. Nevertheless, based on the available data, post-procedural changes in the hs-TnT criterion occurred only in 18% of the patients, and the use of post-procedural ECG and hs-TnT data did not significantly affect the RAISE Score classifications.

Furthermore, since the prevalence of pathological bone scintigraphy results is relatively low, the distribution of patients in subgroup analyses is uneven, which limits the generalizability of the findings. Given the sample size and low event rate, the results must be interpreted with caution, especially regarding prognostic implications. Therefore, our newly proposed simplified screening approaches will once again need to be externally validated, especially in younger patients.

## Conclusion

The prevalence of pathological bone scintigraphy results in AVR patients was 16%, but only half of these patients had a Perugini Score ≥ 2 and a confirmed diagnosis of ATTR-CM. The complex RAISE Score used as screening tool for pathological bone scintigraphy showed a sensitivity of at most 76% when calculated postoperatively, which is a lower performance than reported during derivation and internal validation. Most valuable pre-selection criteria for testing in our cohort included advanced age, CTS, and elevated NT-proBNP levels. The presence of a pathological bone scintigraphy worsened prognosis in AVR patients compared to those with a normal scan. From the upfront clinical perspective, AVR patients ≥ 83 years or those with CTS and/or NT-proBNP ≥ 1,400 pg/mL appear to be appropriate candidates for additional testing with bone scintigraphy, without overstraining resources.

## Supplementary Information

Below is the link to the electronic supplementary material.

ESM 1 DOCX (3.22 MB)

## Data Availability

Data are available from the corresponding author upon reasonable request.
