# Supplementary material for: Cardiac transthyretin amyloidosis in aortic valve replacement: RAISE score performance in the postoperative setting
Source: Clin Res Cardiol. 2025 Nov 4;115(5):826–41. doi: 10.1007/s00392-025-02766-6 (PMC13083505; doi:10.1007/s00392-025-02766-6)
Supplement: Supplementary file 1 — DOCX (3.22 MB) [file 392_2025_2766_MOESM1_ESM.docx]

**Cardiac Transthyretin Amyloidosis in Aortic Valve Replacement: RAISE Score Performance in the Postoperative Setting**

Richard J. Nies^1^ MD, Svenja Ney^1^ MD, Jasper F. Nies^2^ MD, Katharina Seuthe^1^ MD, Jan Grobecker^1^ MD, Friedrich Gruenagel^1^, Stephan Nienaber^1^ MD, Merve Kural^1^ MD, Sascha Macherey-Meyer^1^ MD, Matthieu Schäfer^1^ MD, Clemens Metze^1^ MD, Matti Adam^1^ MD, Maria Papathanasiou^3^ MD, Can Öztürk^4^ MD, Amin Polzin^5^ MD, Fabian Voß^5^ MD, Stephan Baldus^1^ MD, Roman Pfister^1^ MD

^1^ University of Cologne, Faculty of Medicine and University Hospital Cologne, Clinic III for Internal Medicine, Kerpener Str. 62, D-50937 Cologne, Germany

^2^ Department II of Internal Medicine and Center for Molecular Medicine Cologne, University of Cologne, Faculty of Medicine, and University Hospital Cologne, Kerpener Str. 62, D-50937 Cologne, Germany

^3^ University Hospital Frankfurt, Department of Cardiology, Theodor-Stern-Kai 7, 60596, Frankfurt, Germany

^4^ University Hospital Bonn, Heart Centre Bonn, Venusberg-Campus 1, D-53127 Bonn, Germany

^5^ University Hospital Düsseldorf, Heart Centre Düsseldorf, Moorenstr. 5, D-40225 Düsseldorf, Germany

**Short title:** Performance of the RAISE Score after AVR

**- SUPPLEMENTARY MATERIAL -**

Corresponding author:

Dr. med. Richard Nies

University of Cologne, Faculty of Medicine and University Hospital Cologne

Clinic III for Internal Medicine

Kerpener Straße 62

D-50937 Köln, Germany

Phone: +49 221 47876653

Fax: +49 221 47832343

E-mail: richard.nies@uk-koeln.de

**Figure S1:** ROC analysis for age regarding pathological finding in bone scintigraphy.

AUC: 0.841

Optimal cutoff according to Youden Index: 82.6 years

**Figure S2:** ROC analysis for NT-proBNP levels regarding pathological finding in bone scintigraphy.

AUC: 0.775

Optimal cutoff according to Youden Index: 1,433 pg/mL

**Figure S3:** ROC analysis for hsTnT levels regarding pathological finding in bone scintigraphy.

Optimal cutoff according to Youden Index: 0.028 ng/mL

AUC: 0.724

**Figure S4:** ROC analysis for GFR regarding pathological finding in bone scintigraphy.

AUC: 0.593

Optimal cutoff according to Youden Index: 49.5 mL/min

**Figure S5:** ROC analysis for IVSd regarding pathological finding in bone scintigraphy.

Optimal cutoff according to Youden Index: 15.2 mm

AUC: 0.802

**Figure S6:** ROC analysis for E/A ratio regarding pathological finding in bone scintigraphy.

AUC: 0.814

Optimal cutoff according to Youden Index: 0.871

**Figure S7:** ROC analysis for mitral s’ regarding pathological finding in bone scintigraphy.

AUC: 0.768

Optimal cutoff according to Youden Index: 6.5 cm/s

**Figure S8:** All-cause mortality of the entire cohort.

**Figure S9:** All-cause mortality of patients with and without a RAISE Score ≥ 2.


**Figure S10:** All-cause mortality of patients with and without a RAISE Score ≥ 3.

**Figure S11:** All-cause mortality of patients with and without age ≥ 83 years.

**Figure S12:** Hospitalization due to HF of patients with and without a RAISE Score ≥ 2.

**Figure S13:** Hospitalization due to HF of patients with and without a RAISE Score ≥ 3.

**Figure S14:** Hospitalization due to HF of patients with and without age ≥ 83 years.

**Table S1:** Baseline data of patients with pathological bone scintigraphy before AVR, stratified by Perugini Score.

|  | **Perugini Score 1**  **n = 10** | **Perugini Score 2-3**  **n = 11** | **p** |
| --- | --- | --- | --- |
| Subtype of AS  High-gradient; %  Classical low-flow, low-gradient; %  Paradoxical low-flow, low-gradient; %  NYHA class  I; %  II; %  III; %  IV; %  Cardiac device, %  Biomarkers  NT-proBNP (pg/mL); Mdn [Q_1_; Q_3_]  Hs-TnT (ng/mL); Mdn [Q_1_; Q_3_]  LVEF (%); Mdn [Q_1_; Q_3_]  Type of procedure  TAVR, %  SAVR, % | 70.0  10.0  20.0  0.0  10.0  90.0  0.0  10.0  2,630 [1,544; 6,400] (n=9)  0.041 [0.022; 0.057] (n=9)  58 [56; 60]  100.0  0.0 | 72.7  18.2  9.1  9.1  27.3  63.6  0.0  18.2  2,286 [1,700; 5,756] (n=9)  0.051 [0.034; 0.097] (n=8)  57 [55; 62]  100.0  0.0 | 0.709  0.332  0.593  0.965  0.289  0.859  n.a. |

__________________________________________________________________________________________________________________________________________________________________________________

AS = aortic stenosis; Hs-TnT = high-sensitivity troponin T; LVEF = left ventricular ejection fraction; n.a. = not available; NT-proBNP = N-terminal pro-brain natriuretic peptide; NYHA = New York Heart Association; SAVR = surgical aortic valve replacement; TAVR = transcatheter aortic valve replacement.

**Table S2:** Baseline data of patients with pathological bone scintigraphy at 30-day FU after AVR, stratified by Perugini Score.

|  | **Perugini Score 1**  **n = 10** | **Perugini Score 2-3**  **n = 11** | **p** |
| --- | --- | --- | --- |
| **Demographic data**  Male; %  Age (years); Mdn [Q_1_; Q_3_]  BMI (kg/m^2^); Mdn [Q_1_; Q_3_] | 30.0  84 [79; 87]  25.9 [23.1; 31.4] | 90.9  86 [84; 88]  25.6 [24.4; 28.8] | 0.008  0.114  1.000 |
| **Vital parameters**  SBP (mmHg); M ± SD  DBP (mmHg); M ± SD  Heart rate (1/min); Mdn [Q_1_; Q_3_] | 141±22  68±7  68 [61; 80] | 150±20  80±8  74 [64; 79] | 0.319  0.003  0.605 |
| **Symptoms**  NYHA class  I; %  II; %  III; %  IV;%  Decline in NYHA class ≥ I, % | 10.0  80.0  10.0  0.0  90.0 | 36.84  45.5  18.2  0.0  63.6 | 0.249  0.311 |
| **Comorbidities**  Arterial hypertension; %  CAD,%  History of myocardial infarction; %  AF; %  Previous cardiothoracic surgery,%  CKD with dialysis,%  Cardiac device therapy; %  Potential extracardiac manifestations of ATTR  History of CTS; %  Lumbar spinal stenosis; %  Clinical signs of PNP; % | 90.0  70.0  0.0  50.0  0.0  0.0  20.0  40.0  10.0  20.0 | 90.9  36.4  0.0  72.7  0.0  0.0  18.2  27.3  9.1  9.1 | 1.000  0.198  n.a.  0.387  n.a.  n.a.  1.000  0.659  1.000  0.586 |
| **Biomarkers**  Hemoglobin (g/dL); M ± SD  Creatinine (mg/dL); Mdn [Q_1_; Q_3_]  GFR (mL/min); Mdn [Q_1_; Q_3_]  NT-proBNP (pg/mL); Mdn [Q_1_; Q_3_]  Δ NT-proBNP decline (pg/mL); Mdn [Q_1_; Q_3_]  Hs-TnT (ng/mL); Mdn [Q_1_; Q_3_]  Δ hs-TnT decline (ng/mL); Mdn [Q_1_; Q_3_] | 12.1±1.8  1.65 [1.00; 2.00]  33 [23; 58]  1,286 [818; 2,228]  411 [-35; 4762] (n=9)  0.035 [0.020; 0.045]  -0.001 [-0.010; 0.014] (n=9) | 13.1±1.8  1.40 [0.90; 1.50]  49 [41; 75]  1,720 [923; 4,177]  758 [87; 2,223] (n=9)  0.040 [0.028; 0.062]  0.004 [-0.002; 0.022] (n=8) | 0.224  0.072  0.072  0.314  0.863  0.349  0.423 |
| **Electrocardiogram**  Rhythm  Sinus; %  AF*; %  VAT mode or atrial paced; %  Atrioventricular conduction  Normal PQ interval; %  Atrioventricular block I°; %  AF or paced; %  QRS width (ms); Mdn [Q_1_; Q_3_]  QRS configuration  Normal; %  LAHB; %  LBBB; %  RBBB; %  Pacing*, %  QRS-Score (mV)**; M ± SD  Sokolow-Lyon-Index (mV)**; Mdn [Q_1_; Q_3_]  Low voltage; % | 90.0  0.0  10.0  50.0  40.0  10.0  100 [90; 160]  60.0  0.0  20.0  10.0  10.0  11.55±3.31 (n=6)  1.80 [1.40; 2.58] (n=6)  10.0 | 63.6  36.4  0.0  45.5  18.2  36.4  110 [90; 135]  54.5  9.1  9.1  9.1  18.2  13.47±3.63 (n=7)  2.60 [1.80; 3.50] (n=7)  9.1 | 0.074  0.298  0.918  0.805  0.344  0.138  1.000 |
| **Imaging**  IVSd (mm); Mdn [Q_1_; Q_3_]  LVEDD (mm); M ± SD  PWT (mm); Mdn [Q_1_; Q_3_]  RV free wall thickness (mm); Mdn [Q_1_; Q_3_]  LVMI (g/m^2^); Mdn [Q_1_; Q_3_]  LVEF (%); Mdn [Q_1_; Q_3_]  GLS (%) **; M ± SD  Apical sparing **; %  Apical sparing index**; Mdn [Q_1_; Q_3_]  LAVI (mL/m^2^); Mdn [Q_1_; Q_3_]  SVI (mL/m^2^); Mdn [Q_1_; Q_3_]  FAC (%); Mdn [Q_1_; Q_3_]  TAPSE (mm); Mdn [Q_1_; Q_3_]  RA area (cm^2^); Mdn [Q_1_; Q_3_]  RVEDD (mm); Mdn [Q_1_; Q_3_]  AV v_max_ (m/s); M ± SD  AV dP_max_ (mmHg); Mdn [Q_1_; Q_3_]  AV dP_mean_ (mmHg); Mdn [Q_1_; Q_3_]  AVA (cm^2^); Mdn [Q_1_; Q_3_]  PVL ≥ moderate; %  E/A; Mdn [Q_1_; Q_3_]  E _max_ (cm/s); Mdn [Q_1_; Q_3_]  A _max_ (cm/s); M ± SD  DT (ms); M ± SD  Septal e‘ (cm/s); Mdn [Q_1_; Q_3_]  Lateral e‘ (cm/s); Mdn [Q_1_; Q_3_]  Mitral s‘ (cm/s); Mdn [Q_1_; Q_3_]  E/e’; Mdn [Q_1_; Q_3_]  TR v_max_ (m/s); Mdn [Q_1_; Q_3_]  TR dP_max_ (mmHg); Mdn [Q_1_; Q_3_]  sPAP (mmHg); Mdn [Q_1_; Q_3_]  Severe MS, %  Severe MR, %  Severe TR, %  Pericardial effusion; % | 14 [13; 15]  46±6  10 [8; 11]  7 [5; 7]  108 [81; 117]  58 [56; 62]  -19.0±4.1 (n=6)  0.0 (n=6)  0.85 [0.56; 0.91] (n=4)  35 [29; 40]  40 [32; 49]  45 [40; 46]  21 [19; 24]  16 [13; 18]  35 [27; 39]  1.86±0.47  14 [10; 17]  7 [5; 10]  1.66 [1.52; 2.25]  0.0  0.83 [0.72; 1.19]  87 [68; 123]  103±38  279±115  5 [4; 6] (n=9)  6 [6; 10] (n=9)  7 [7; 8] (n=9)  12.1 [9.8; 21.8] (n=9)  2.9 [2.5; 3.3]  32.5 [24.0; 45.3]  35.5 [27.0; 48.3]  0.0  0.0  0.0  0.0 | 17 [15; 18]  47±7  14 [13; 17]  9 [8; 10]  147 [129; 164]  57 [55; 60]  -13.9±2.8 (n=7)  85.7 (n=7)  1.16 [0.85; 1.67] (n=7)  48 [40; 60]  41 [26; 45]  46 [39; 49] (n=10)  22 [14; 23]  25 [19; 27]  37 [34; 39]  2.25±0.55  22 [11; 28]  13 [8; 15]  1.88 [1.60; 2.10]  18.2  1.30 [0.87; 2.15] (n=7)  91 [73; 121]  85±38 (n=7)  255±92  6 [4; 7]  8 [7; 10]  6 [5; 7]  16.0 [9.9; 19.6]  2.8 [2.7; 2.9]  30.0 [28.0; 35.0]  34.0 [31.0; 40.0]  0.0  0.0  0.0  0.0 | 0.008  0.674  0.001  0.001  0.020  0.654  0.021  0.005  0.164  0.002  0.705  0.631  0.756  0.001  0.282  0.094  0.282  0.197  0.918  0.365  0.193  0.863  0.348  0.594  0.552  0.131  0.025  0.766  0.756  0.605  0.918  n.a.  n.a.  n.a.  n.a. |
| **Scores**  RAISE-Score ≥ 2  RAISE Score ≥ 3  H2FPEF Score ≥ 5 | 70.0  50.0  80.0 | 81.8  72.7  90.9 | 0.635  0.387  0.586 |
| **Amyloidosis diagnostic**  Serum immunofixation  No MGUS; %  MGUS; %  Inconclusive; %  Not done; %  EMB, %  Normal finding  Pathological finding | 70.0  20.0  10.0  0.0  20.0  100.0  0.0 | 100.0  0.0  0.0  0.0  0.0  n.a.  n.a. | 0.146  0.214  n.a.  n.a. |
| **ATTR-CM**  None, %  ATTR-CM, %  Not classifiable due to missing EMB, %  **ATTR specific medication**  Tafamidis 61mg; % | 20.0  0.0  80.0  0.0 | 0.0  100.0  0.0  100.0 | <0.001  <0.001 |

*2 patients with atrial fibrillation and ventricular pacing; **only patients without ventricular pacing and complete bundle branch block; AF = atrial fibrillation; ATTR = transthyretin amyloidosis; ATTR-CM = transthyretin amyloidosis cardiomyopathy; AV = aortic valve; AVA = aortic valve area; BMI = body mass index; CAD = coronary artery disease; CKD = chronic kidney disease; CTS = carpal tunnel syndrome; DBP = diastolic blood pressure; dP = differential pressure; DT = deceleration time; EMB = endomyocardial biopsy; FAC = fractional area change; GFR = glomerular filtration rate; GLS = global longitudinal strain; Hs-TnT = high-sensitivity troponin T; IVSd = interventricular septum thickness at end-diastole; LAHB = left anterior hemiblock; LAVI = left atrial volume index; LBBB = left bundle branch block; LVEDD = left ventricular end-diastolic diameter; LVEF = left ventricular ejection fraction; LVMI = left ventricular mass index; M = mean; MAPSE = mitral annular plane systolic excursion; Mdn = median; MGUS = monoclonal gammopathy with undetermined significance; MR = mitral regurgitation; MS = mitral stenosis; n.a. = not available; NT-proBNP = N-terminal pro-brain natriuretic peptide; NYHA = New York Heart Association; PNP = polyneuropathy; PVL = paravalvular leak; PWT = posterior wall thickness; Q = quartile; RA = right atrium; RBBB = right bundle branch block; RV = right ventricular; RVEDD = right ventricular end-diastolic diameter; SBP = systolic blood pressure; SD = standard deviation; sPAP = systolic pulmonary arterial pressure; SVI = stroke volume index; TAPSE = tricuspid annular plane systolic excursion; TR = tricuspid regurgitation; v = velocity; VAT mode = pacemaker senses electrical signals from the atrium and stimulates the ventricle to maintain synchrony between the atrium and ventricle.

**Table S3:** Patients with definitive diagnosis of ATTR-CM.

| **ATTR-CM** | **Age (y)** | **AF** | **History of CTS** | **Creatinine (mg/dL)** | **GFR (mL/min)** | **NT-proBNP (pg/mL)** | **Hs-TnT (ng/mL)** | **IVSd (mm)** | **Apical sparing** | **MAPSE (mm)** | **E/A** | **Mitral s‘** |
| --- | --- | --- | --- | --- | --- | --- | --- | --- | --- | --- | --- | --- |
| Patient 1 | 84 | Yes | No | .90 | 75 | 2300 | .015 | 19 | Yes | 9 | .70 | 7 |
| Patient 2 | 86 | No | No | .80 | 80 | 923 | .017 | 16 | No | 12 | 1.01 | 7 |
| Patient 3 | 86 | No | Yes | 1.40 | 49 | 465 | .034 | 15 | Yes | 14 | 1.51 | 6 |
| Patient 4 | 84 | No | No | 1.00 | 68 | 1720 | .049 | 18 | Yes | 9 | 1.30 | 4 |
| Patient 5 | 83 | Yes | Yes | .70 | 86 | 692 | .028 | 17 | Yes | 12 | AF | 6 |
| Patient 6 | 86 | Yes | Yes | 1.40 | 44 | 4177 | .036 | 17 | No | 9 | AF | 6 |
| Patient 7 | 88 | Yes | No | 1.30 | 49 | 1519 | .040 | 20 | n.a. | 11 | AF | 7 |
| Patient 8 | 88 | Yes | No | 1.80 | 32 | 5365 | .069 | 19 | Yes | 9 | AF | 6 |
| Patient 9 | 88 | Yes | No | 1.50 | 41 | 2235 | .097 | 13 | Yes | 8 | 2.66 | 4 |
| Patient 10 | 85 | Yes | No | 1.50 | 32 | 6143 | .061 | 14 | Yes | 7 | 2.15 | 4 |
| Patient 11 | 86 | Yes | No | 1.50 | 42 | 1435 | .062 | 16 | No | 18 | .87 | 3 |

AF = atrial fibrillation; ATTR-CM = transthyretin amyloidosis cardiomyopathy; CTS = carpal tunnel syndrome; GFR = glomerular filtration rate; Hs-TnT = high-sensitivity troponin T; IVSd = interventricular septum thickness at end-diastole; MAPSE = mitral annular plane systolic excursion; n.a. = not available; NT-proBNP = N-terminal pro-brain natriuretic peptide.

**Table S4:** Screening performance of IVSd ≥ 15 mm regarding pathological bone scintigraphy (**A**) and definitive diagnosis of ATTR-CM (**B**).

| **A** | **IVSd ≥ 15 mm** | **IVSd < 15 mm** |  |
| --- | --- | --- | --- |
| **Perugini Score 0** | n = 29 | n = 81 | **Specificity: 74%** |
| **Perugini Score 1-3** | n = 10 | n = 11 | **Sensitivity: 48%** |
|  | **PPV: 26%** | **NPV: 88%** |  |

| **B** | **IVSd ≥ 15 mm** | **IVSd < 15 mm** |  |
| --- | --- | --- | --- |
| **No/unconfirmed ATTR-CM** | n = 30 | n = 90 | **Specificity: 75%** |
| **ATTR-CM** | n = 9 | n = 2 | **Sensitivity: 82%** |
|  | **PPV: 23%** | **NPV: 98%** |  |

**Table S5:** Screening performance of NT-proBNP ≥ 1,400 pg/mL regarding pathological bone scintigraphy (**A**) and definitive diagnosis of ATTR-CM (**B**).

| **A** | **NT-proBNP ≥ 1,400 pg/mL** | **NT-proBNP < 1,400 pg/mL** |  |
| --- | --- | --- | --- |
| **Perugini Score 0** | n = 25 | n = 85 | **Specificity: 77%** |
| **Perugini Score 1-3** | n = 13 | n = 8 | **Sensitivity: 62%** |
|  | **PPV: 34%** | **NPV: 91 %** |  |

| **B** | **NT-proBNP ≥ 1,400 pg/mL** | **NT-proBNP < 1,400 pg/mL** |  |
| --- | --- | --- | --- |
| **No/unconfirmed ATTR-CM** | n = 30 | n = 90 | **Specificity: 75%** |
| **ATTR-CM** | n = 8 | n = 3 | **Sensitivity: 73%** |
|  | **PPV: 21%** | **NPV: 97%** |  |
